# FGF9-induced proliferative response to eosinophilic inflammation in oesophagitis

**DOI:** 10.1136/gut.2008.157628

**Published:** 2008-10-31

**Authors:** D J Mulder, I Pacheco, D J Hurlbut, N Mak, G T Furuta, R J MacLeod, C J Justinich

**Affiliations:** 1 Department of Anatomy and Cell Biology, Queen’s University, Kingston, Ontario, Canada; 2 Gastrointestinal Diseases Research Unit, Kingston General Hospital, Kingston, Ontario, Canada; 3 Department of Pathology and Molecular Medicine, Queen’s University, Kingston, Ontario, Canada; 4 Section of Gastroenterology, Hepatology and Nutrition, The Children’s Hospital Denver, University of Colorado Denver, Aurora, Colorado, USA; 5 Departments of Physiology and Medicine, Queen’s University, Kingston, Ontario, Canada; 6 Pediatric Gastroenterology, Departments of Pediatrics, Anatomy and Cell Biology, Physiology and Medicine, Queen’s University, Kingston, Ontario, Canada

## Abstract

**Background:**

Oesophagitis is characterised by basal cell hyperplasia and activated eosinophils, which release mediators including major basic protein (MBP). MBP and its mimetic polyarginine activate the calcium sensing receptor (CaSR) on oesophageal epithelium. Fibroblast growth factor 9 (FGF9) is implicated in epithelial homeostasis and proliferative response to injury, but has not been characterised in the oesophagus.

**Objective:**

To characterise FGF9 in oesophageal epithelium and oesophagitis, as the result of MBP activation of the CaSR.

**Methods:**

Human oesophageal epithelial cells (HET-1A) were used to compare affects of calcium, polyarginine and MBP-peptide on FGF9. HET-1A were transfected with interfering RNA (siRNA^CaSR^). FGF9, FGF receptors 2 and 3, bone morphogenetic protein (BMP)-2, BMP-4 and noggin mRNA expression were detected by reverse transcriptase polymerase chain reaction. FGF9 was measured from HET-1A and from normal, gastro-oesophageal reflux and eosinophilic oesophagitis (EoE) patient biopsies using ELISA and immunohistochemistry. HET-1A proliferation was studied using bromodeoxyuridine and MTT.

**Results:**

FGF9 was secreted by HET-1A cells treated with polyarginine and MBP-peptide, but not calcium. This effect was abrogated by siRNA^CaSR^. FGF9 receptor mRNA was present. HET-1A cells proliferated following rhFGF9, but not MBP-peptide treatment, and rhFGF9 altered transcription of downstream proliferation-related genes (noggin, BMP-2 and BMP-4). FGF9 was increased in biopsies from patients with eosinophilic oesophagitis, which correlated with basal hyperplasia.

**Conclusion:**

Eosinophil-released MBP acts on the CaSR to increase FGF9 in oesophageal epithelial cells, leading to proliferation. Increased FGF9 is found in biopsies of EoE patients and may play a role in the pathogenesis of oesophagitis.

Oesophagitis is characterised by inflammation and morphological changes to the epithelium including basal zone hyperplasia and elongation of vascular papillae. Until recently, gastro-oesophageal reflux disease (GORD) has been the primary disease associated with oesophageal mucosal inflammation. During the last decade an emerging body of literature supports the appearance of a new disease termed eosinophilic oesophagitis (EoE). A systematic review of the literature was recently published as a consensus statement, and EoE was defined by 15 or more intraepithelial eosinophils per high-power field (HPF) in the oesophageal mucosa.^1^ In EoE, eosinophils degranulate, releasing inflammatory mediators, the most abundant of which is major basic protein (MBP).^2^ ^3^ MBP was originally defined as a cytotoxin.^4^ More recent studies show that MBP acts as a mediator of inflammation and can alter the function of epithelium.^5^^–^7 In addition, biopsies from patients with EoE and murine models of this disease demonstrate high levels of eosinophilic inflammation and also marked basal zone hyperplasia.^8^ ^9^ The pathogenetic link between these two histological findings is unknown. We postulate that MBP released from eosinophils acts on oesophageal epithelial cells to cause these changes.

There is no known receptor for MBP. Polyarginine is similar in structure and function to the biologically active moiety of MBP and has been used as a molecular mimic for MBP. Poly-l-arginine is a ligand for the extracellular calcium sensing receptor (CaSR),^10^ a G-protein coupled receptor, which is found on many cell types throughout the body.^11^ The CaSR has many known ligands, including calcium, other polyvalent cations, spermine and amyloid β-peptide.^11^ We have previously characterised the presence and functional activity of the CaSR in the human oesophageal epithelium and in the oesophageal epithelial cell line HET-1A.^12^ Since the actions of MBP mimic polyarginine, we hypothesise that MBP, released by eosinophils in oesophagitis, acts as a CaSR ligand.

In order to study the effects of MBP in oesophageal epithelium, synthetic peptides of the active moiety of MBP are used. These synthetic peptides of MBP have been shown to mimic the actions of MBP.^13^ Treatment of cultured cells with synthetic peptides, such as an MBP-peptide, is a suitable model for assessing alterations in gene expression.^14^ Array analysis of HET-1A cells treated with Ca^2+^ (2.5 mmol/l) or MBP-peptide (5 μmol/l) demonstrated increased fibroblast growth factor 9 (FGF9) mRNA only after MBP-peptide stimulation (supplemental material). FGF9 is a 26 kDa secreted protein, which leads to epidermal proliferation and wound healing in the skin of mice^15^ and has a paracrine role in toxic liver injury.^16^ FGF9 acts on its receptors (FGFR2 and 3) to activate intracellular signal transduction cascades on target cells,^17^ ^18^ which affects the expression of downstream target genes including members of the bone morphogenetic protein (BMP) family. The expression of BMP-2, BMP-4 and noggin (the inducible antagonist of BMP-2) are known to affect proliferation and cell survival as a result of FGF9 activation in human tissue.^19^ BMP-4 is a downstream target of FGF and canonical Wnt signalling that can induce proliferation.^20^ We have shown that BMP-2 is a CaSR-dependent factor important for barrier function in T-84 colonic epithelial cells.^21^ Decreasing BMP-2 expression may decrease apoptosis of oesophageal epithelial cells. Noggin acts as an antagonist of BMP-2 and is known to induce proliferation in colonic epithelial cells.^21^ ^22^ Since functional CaSR is present on oesophageal epithelium, this suggests that MBP-induced FGF9 participates in epithelial hyperplasia in oesophagitis.

Eosinophil-derived products in oesophagitis lead to increased FGF9 secretion by oesophageal epithelial cells. Alterations to the oesophageal mucosa, including basal zone hyperplasia and vascular papillae elongation, should correlate with FGF9 protein concentrations in mucosal biopsies. In this study we demonstrate induction of FGF9 mRNA and protein expression following activation of the CaSR by MBP-peptide and polyarginine but not calcium. FGF9 increases mRNA in HET-1A cells for proliferative BMP signalling and leads to proliferation as measured by in vitro assays. Patients with EoE have increased FGF9 in oesophageal mucosal biopsies, which correlates positively with alterations in histology known to occur with oesophagitis. These results suggest that FGF9 may be a key growth factor in the pathophysiology of EoE.

## MATERIALS AND METHODS

### Cell culture

The HET-1A human oesophageal epithelial cell line was obtained from ATCC (Manassas, Virginia, USA), and cultured according to the original protocol.^23^ Cells were passaged weekly and studied between passages 39 and 45. Bronchial epithelial growth media (BEGM) with 0.5 mmol/l calcium chloride was used as control media. Poly-l-arginine (MW = 5000–15 000; Sigma–Aldrich, Oakville, Ontario, Canada) was added at a concentration of 1.3 μmol/l. MBP-peptide (0.1, 0.5 and 5 μmol/l) was synthesised as described below. Calcium (2.5 mmol/l) and recombinant human FGF9 (rhFGF9, 20–40 ng/ml; Abcam, Cambridge, Massachusetts, USA) were also used to treat to HET-1A cells where indicated.

### Human tissue biopsies

Grasp biopsies of the distal oesophagus were obtained from patients undergoing endoscopy for evaluation of gastrointestinal symptoms. Biopsies were fixed in 10% formaldehyde and embedded in paraffin for routine histopathology; adjacent biopsies were homogenised for FGF9 measurement. Patient samples with a basal zone ⩽25% were considered normal. Patients with a basal zone of >25% and/or elongated vascular papillae and 0–14 eosinophils per high-power field (HPF, ×40 objective) were considered to have gastro-oesophageal reflux disease (GORD). Eosinophilic oesophagitis (EoE) was defined as the presence of 15 or more eosinophils in the maximally affected HPF, where GORD had been ruled out.^1^ Basal hyperplasia >25% was a feature seen in all EoE biopsies.

### Measurement of fibroblast growth factor 9

Tissue biopsies from patients for FGF9 protein analysis was homogenised in 600 μl of phosphate-buffered saline (PBS) and then centrifuged for 20 min at 2000 *g* at 4°C and supernatants were collected for enzyme-linked immunosorbent assay (ELISA) and protein measurement. For cell culture experiments, HET-1A cells were plated in equal density in 24-well plates and supernatants were collected at 18 h following treatment. Total protein concentration was determined by the Bradford method and FGF9 levels were measured by ELISA (Ray BioTech, Norcross, Georgia, USA) according to the manufacturer’s instruction, with results expressed as pg of FGF9/ml/mg of total protein.

### Immunohistochemistry

Formalin-fixed, paraffin-embedded oesophageal mucosal biopsies sectioned at 5 μm were rehydrated and endogenous peroxidase activity was quenched with 3% H_2_O_2_. Non-specific binding was blocked by 30 min incubation with 5% goat serum/0.02% azide/0.02% Tween-20 in PBS. Primary mouse anti-human immunoglobulins were purchased from Santa Cruz Biotechnology (anti-FGF9 and anti-FGFR3; Santa Cruz, California, USA) and R&D Systems (anti-FGFR2; R&D Systems, Minneapolis, Minnesota, USA). Slides were incubated overnight with anti-FGF9 (1:50), anti-FGFR2 (1:20) or anti-FGFR3 (1:50) or with normal mouse immunoglobulin G (IgG) as a negative control. Slides were then treated with biotinylated anti-mouse IgG secondary antibody and then horseradish peroxidase–streptavidin complex each for 30 min (Santa Cruz Biotechnology). Slides were stained using the avidin–biotin–peroxidase complex (ABC) method with 3,3′-diaminobenzidine substrate, counterstained with haematoxylin and visualised using a Nikon Eclipse TE-2000U microscope, a Nikon DXM 1200C digital camera (Nikon, Mississauga, Ontario, Canada) and NIS-Elements BR 2.30 software.

### Synthesis of biologically active major basic protein-peptide

A peptide consisting of amino acids 89–117 of MBP (H-Gly-Gly-His-Cys-Val-Ala-Leu-Cys-Thr-Arg-Gly-Gly-Tyr-Trp-Arg-Arg-Ala-His-Cys-Leu-Arg-Arg-Leu-Pro-Phe-Ile-Cys-Ser-Tyr-OH)^13^ was synthesised by the Queen’s Protein Discovery and Function Unit. This peptide was generated from 0.2 mmol/l 4-(2′,4′-dimethoxyphenyl-fmoc-aminomethyl)-phenoxy resin from 9-fluoroenylmethoxycarbonyl l-amino acid derivatives using an ACT 350 automated multiple peptide synthesiser (Advanced Chemtech, Louisville, Kentucky, USA).

### Generation and transfection of short interfering RNA

A sequence matching the criteria to be a target of siRNA against the CaSR gene, but against no other gene, was determined by BLAST analysis. This sequence is located between nucleotides 371 and 390 in the CaSR gene (5′-AACCTTGATGAGTTCTGCAAC-3′). Sense and antisense template DNA primers were generated and siRNA was transcribed following the manufacturer’s protocol (Silencer siRNA Construction Kit; Ambion, Austin, Texas, USA). A control siRNA construct, consisting of a scrambled sequence of the same nucleotides, was also synthesised. HET-1A cells were transfected with 750 pmol/l siRNA^CaSR^ or scrambled siRNA using Superfect reagent (3 mg/ml; QIAGEN, Valencia, California, USA). We have shown previously that transfection using siRNA^CaSR^ significantly reduces the expression and functional activity of the CaSR in HET-1A cells.^12^ ^21^

### Reverse transcriptase polymerase chain reaction analysis

RNA was isolated from HET-1A cells using Trizol (Invitrogen, Carlsbad, California, USA). Reverse transcription was performed using the QIAGEN RT Kit (QIAGEN) and the QiaQuick PCR Kit (QIAGEN) as previously described.^12^ Primers with specific annealing temperatures and cycles included: FGF9 (5′-AAG GAC TGC GGC CTG ATG-3′ and 5′-TTT GCT TTA AGT TCA CTG CGA TG-3′) at 59.0°C for 33 cycles; GAPDH (5′-TTA GCA CCC CTG GCC AAG G-3′ and 5′-CTT ACT CCT TGG AGG CCA TG-3′) at 55.0°C for 24 cycles; FGFR2 (5′-AAC CTA GCT ACA CTG AGC AGG G-3′ and 5′-TTC TCC TCC TGG GGA AGA TT-3′) at 60.5°C for 32 cycles; and FGFR3 (5′-CGG AAA GTT CGT CGC TGG-3′ and 5′-TTA CTG GGC CCT GAG TCT GG-3′) at 60.5°C for 32 cycles. Standard conditions for each PCR cycle were 94°C for 30 s, followed by specific primer annealing temperature for 60 s and 72°C for 30 s. Reverse transcriptase polymerase chain reaction (RT-PCR) using BMP-2, BMP-4 and noggin primers and conditions was performed as we have previously described.^21^

### Cellular proliferation assays

Proliferation assays were performed using the CellTiter 96 Non-Radioactive Cell Proliferation Assay Kit (Promega, Madison, Wisconsin, USA) and the BrdU Cellular Proliferation Assay Kit (Roche, Laval, Quebec, Canada) following the manufacturer’s instructions. Cells were plated at a concentration of 5×10^4^ cells/ml. After 48 h, culture media was removed and replaced with control media (BEGM) or media supplemented with 20 or 40 μg/ml recombinant human FGF9 (rhFGF9; Abcam), 0.1, 0.5 or 5 μmol/l MBP-peptide or 10% FCS and incubated for 24–48 h as indicated. At each endpoint, the optical density of each solution was measured at 570 nm and 450 nm for MTT conversion and BrdU incorporation, respectively. (MTT is 3-(4,5-dimethylthiazol-2-yl)-2,5-diphenyltetrazolium bromide.)

### Histopathology

Haematoxylin, phloxine and saffron (HPS)-stained 5 μm thick sections of the formalin-fixed and paraffin-embedded oesophageal mucosal biopsies were reviewed by a pathologist (DJH) who was blinded to patient and clinical diagnosis. Biopsies of patients were used when tissue was well-oriented and multiple sections examined, with the most representative chosen for analysis. Slides were assessed for the following histological parameters:

- basal zone hyperplasia, defined as normal (⩽25%; 1), mild (26–50%; 2), moderate (51–75%; 3) or severe (>75%; 4);^24^
- degree of extension of vascular papillae of lamina propria into squamous epithelium, defined as normal (⩽50% epithelial thickness; 1) or elongated (>50%; 2);^1^
- maximum intraepithelial eosinophil number per HPF (×40 objective).^1^

Tabulated scores represent the mean (with the standard error) of seven patients in each group. Maximum eosinophils/HPF is represented as the mean with the standard error (range of eosinophils).

### Statistics and calculations

Results are presented for quantitative experiments as the mean of the number of experiments (n) with error bars representing the standard error of the mean. Experimental results that are visually represented are the result of three separate and consistent experiments where one representative image is shown. Statistical analysis was performed using Graphpad Prism software V5.0. Significance (p<0.05) for quantitative data was calculated using an unpaired, two-tailed Student t test for two-group comparison and a one-way analysis of variance (ANOVA) was used to compare experiments containing more than two groups. For comparison of data from patient biopsies a non-parametric Kruskal–Wallis test followed by a Dunn’s multiple comparison test was used to determine significance.

## RESULTS

### Effect of major basic protein peptide and polyarginine on CaSR-mediated FGF9 transcript expression and secretion

**Figure 1:**
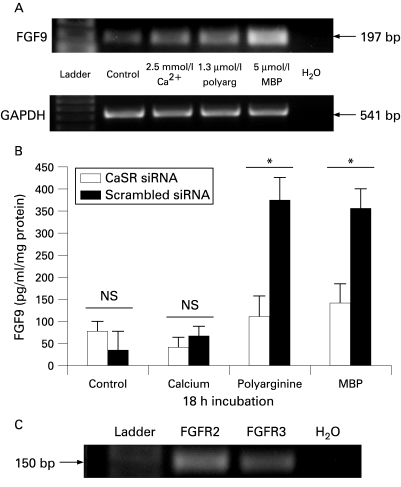
Fibroblast growth factor 9 (FGF9) is increased by major basic protein (MBP)-peptide or polyarginine. (A) Reverse transcriptase polymerase chain reaction (RT-PCR) of mRNA from human oesophageal epithelial (HET-1A) cells. Increased FGF9 mRNA following 18 h incubation in control media, 2.5 mmol/l calcium chloride, 1.3 μmol/l polyarginine or 5 μmol/l MBP-peptide, with glyceraldehyde-3-phosphate dehydrogenase (GAPDH) as an internal control and water substituted for cDNA as a negative control. A representative image is shown (n = 3). (B) FGF9 protein detection by enzyme-linked immunosorbent assay. FGF9 protein from HET-1A cell supernatants after 18 h of incubation in control media, 2.5 mmol/l calcium chloride, 1.3 μmol/l polyarginine or 5 μmol/l MBP-peptide. Cells transfected with siRNA^CaSR^ (open bars) or scrambled siRNA constructs (solid bars). n = 3; NS, not significant; *p<0.05. (C) Identification of mRNA for both biologically active FGF9 receptors, FGFR2 and FGFR3 (all isoforms), in HET-1A oesophageal epithelial cells. Detected by RT-PCR, a representative image is shown (n = 3).

RT-PCR was performed on cDNA from HET-1A oesophageal epithelial cells incubated for 18 h in calcium (2.5 mmol/l), polyarginine (1.3 μmol/l) or MBP-peptide (5 μmol/l). Bands representing reverse transcribed FGF9 mRNA were observed at the expected 197 bp length. Compared to control conditions, expression of FGF9 mRNA was only slightly greater in cells treated with calcium but much greater with polyarginine and MBP-peptide treatment (fig 1A, n = 3), with consistent expression of housekeeping gene GAPDH. Therefore, constitutive FGF9 mRNA expression was increased by both polyarginine and MBP-peptide.

HET-1A cells transiently transfected with short interfering (si)RNA^CaSR^ or with scrambled sequence of siRNA^CaSR^ were treated with control media, increased calcium (2.5 mmol/l), polyarginine (1.3 μmol/l) or MBP-peptide (5 μmol/l) for 18 h and ELISA was used to measure FGF9 secretion (fig 1B, n = 3). Low levels of FGF9 were secreted constitutively by HET-1A cells and were not affected by siRNA^CaSR^ transfection. Control media (0.1 mmol/l Ca^2+^) and increased calcium (2.5 mmol/l) treatment did not show significantly different FGF9 production following transfection of siRNA^CaSR^ compared to scrambled siRNA transfection. Treatment with polyarginine and MBP-peptide increased FGF9 secretion from HET-1A cells. Transfection with siRNA^CaSR^ abrogated the production of FGF9 from polyarginine and MBP-peptide treated cells compared with scrambled siRNA transfected cells demonstrating that this is a CaSR-dependent process.

### FGF receptor 2 and 3 mRNA expression in HET-1A cells

To investigate the possibility that FGF9 can act in an autocrine manner through FGF receptors on HET-1A cells, RT-PCR of isolated mRNA identified FGF receptor isoforms 2 and 3. A transcript for both FGFR2 and 3 was detected at the expected lengths of 154 bp and 150 bp, respectively (fig 1C, n = 3).

### FGF9 alters transcription of BMP genes

**Figure 2:**
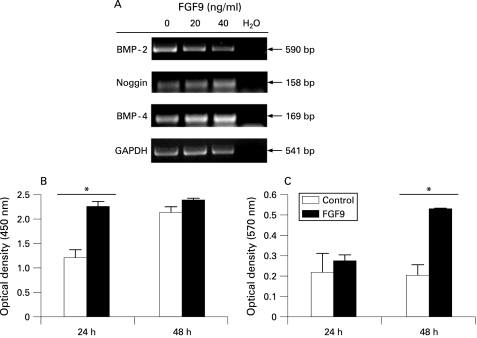
(A) Treatment of human oesophageal epithelial (HET-1A) cells for 40 h with 20 or 40 ng/ml recombinant human fibroblast growth factor (rhFGF9) results in decreased bone morphogenetic protein-2 (BMP-2) mRNA and increased mRNA for noggin and BMP-4. Cells were analysed by reverse transcriptase polymerase chain reaction. Glyceraldehyde-3-phosphate dehydrogenase (GAPDH) was used as an internal control and water as a negative control. A representative image of one of three separate experiments is shown. (B) Bromodeoxyuridine (BrdU) assay. HET-1A cells incubated with rhFGF9 (20 ng/ml) supplemented media (solid bars) show an increase in proliferation compared with cells grown in control media (open bars), at 24 h. n = 3, *p<0.05. (C) MTT assay. Proliferation of HET-1A cells in control media (open bars) and cells in rhFGF9 (20 ng/ml) supplemented media (solid bars). An increase in proliferation with FGF9 was detected at 48 h. n = 3, *p<0.05. (MTT is 3-(4,5-dimethylthiazol-2-yl)-2,5-diphenyltetrazolium bromide.)

Transcript isolated from HET-1A cells treated for 40 h in control media or media supplemented with 20 or 40 ng/ml recombinant human (rh)FGF9 protein was analysed by RT-PCR and gel electrophoresis. Increased rhFGF9 induced a concentration dependent decrease in BMP-2 transcripts and increase in both noggin and BMP-4 mRNA expression. The experiment was performed in triplicate and a representative gel is shown in fig 2A.

### FGF9, but not MBP-peptide, stimulates HET-1A cell proliferation

BrdU and MTT assays were performed on subconfluent HET-1A cells in the presence and absence of rhFGF9 (20 ng/ml). BrdU incorporation in rhFGF9 treated cells was significantly increased at 24 h (p<0.05) indicating proliferation, as shown in fig 2B (n = 3). The same experimental conditions were analysed by MTT assay; after 48 h of rhFGF9 treatment the presence of MTT significantly increased (p<0.05, n = 3; fig 2C).

**Figure 3:**
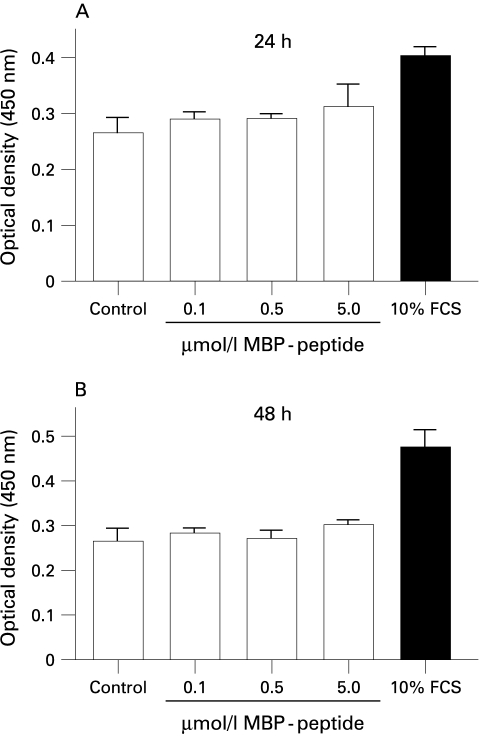
Proliferation of human oesophageal epithelial (HET-1) cells as determined by bromodeoxyuridine (BrdU) at 24 and 48 h. Cells were cultured in control media or media supplemented with major basic protein (MBP)-peptide (0.1, 0.5 or 5 μmol/l) or 10% fetal calf serum (FCS). FCS acted as a positive control, no significant MBP-peptide induced proliferation was observed (n = 3).

BrdU incorporation was analysed following treatment of HET-1A cells with 0.1, 0.5 and 5 μmol/l MBP-peptide for 24 or 48 h. BEGM supplemented with 10% FCS was used as a positive control for proliferation. Proliferation of HET-1A cells was not significantly altered at either time by the addition to MBP-peptide to culture medium (n = 3; fig 3A,B).

### Patients

The present study includes 21 paediatric patients who underwent upper endoscopy for a variety of symptoms. Three groups were distinguished by histopathology: normal oesophagus (n = 7), GORD (n = 7) or EoE (n = 7), with an average age of 10.4, 12.9 and 9.1 years, respectively (table 1). Five of seven patients with EoE (71.4%) were male. One normal patient was described as having friability, but no inflammation was seen on biopsy. Endoscopy of patients with GORD was normal, appearing in four cases (57.1%), and few eosinophils, other inflammatory cells or basal change was seen. Only patients with EoE presented with symptoms of dysphagia. All EoE patients had abnormal endoscopic features (most commonly linear furrowing in six patients) and biopsies containing ⩾15 eosinophils/HPF and basal zone hyperplasia.

**Table 1:** Patient characteristics

Diagnosis	Age (years)	Sex	Symptom	Atopy	Endoscopy	Maximal eosinophils/high-power field	Medication
Normal							
1	16	F	Vomiting	No	Normal	0	None
2	2	M	Feeding aversion	Asthma	Normal	0	Inhaled steroids*
3	11	F	Pain, vomiting	No	Normal	0	None
4	14	M	Pain, vomiting	No	Normal	0	Proton pump inhibitor
5	10	F	Pain, vomiting	No	Normal	0	None
6	16	F	Pain, vomiting	Asthma	Friability	0	Proton pump inhibitor
7	4	M	Pain, vomiting	Asthma	Normal	0	None
Gastro-oesophageal reflux disease							
1	16	F	Pain	No	Normal	2	None
2	14	M	Pain	No	Furrowing, nodularity	9	Proton pump inhibitor
3	17	F	Pain	No	Normal	0	Proton pump inhibitor
4	14	M	Diarrhoea, vomiting	Asthma, food allergies	Furrowing, concentric rings	0	Inhaled steroids*
5	9	F	Vomiting	No	Erosions	0	Metoclopramide
6	14	F	Pain, vomiting	Asthma	Normal	0	Proton pump inhibitor
7	6	F	Diarrhoea, vomiting	No	Normal	0	Metoclopramide
Eosinophilic oesophagitis							
1	13	F	Dysphagia	No	Furrowing, concentric rings	271	None
2	4	M	Dysphagia	Asthma, food allergies	Furrowing	160	Inhaled steroids*
3	8	M	Pain	No	Furrowing, papules	18	None
4	16	M	Dysphagia	Asthma, environmental allergies	Furrowing, concentric rings	63	Cetirizine
5	8	M	Dysphagia	Allergic rhinitis	Furrowing, friability	114	None
6	11	F	Dysphagia	Asthma, food allergy	Papules	169	Proton pump inhibitor, inhaled steroids*
7	4	M	Vomiting	Food allergy	Furrowing	234	None

*For asthma.

### FGF9 is associated with eosinophilic oesophagitis

**Figure 4:**
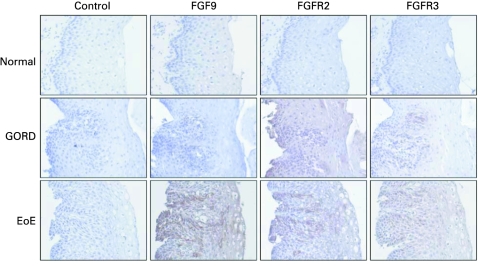
Localisation of fibroblast growth factor 9 (FGF9) and FGF receptors 2 and 3 by immunohistochemistry in histologically normal, gastro-oesophageal reflux disease (GORD) and eosinophilic oesophagitis (EoE) oesophageal mucosal biopsies. Negative control (left side panels) mouse immunoglobulin G (IgG) substituted for primary antibody. Brown colour indicates positive staining for all primary antibodies. FGF9 and receptor immunostaining was increased in oesophagitis and was most pronounced in EoE (n = 3; magnification, ×400).

FGF9, FGFR2 and FGFR3 were localised using immunohistochemistry (n = 3, fig 4). Expression of FGF9 was observed in biopsies from patients with GORD and to a greater extent EoE, compared to normal control biopsies. Staining for FGF receptor 2 (FGFR2), the major epithelial FGFR, was observed throughout the epithelium in biopsies from patients with GORD and EoE; FGFR3 staining appears less prominent. In patients with EoE, epithelial cells residing in the most basal layers of the epithelium and those immediately adjacent to vascular papillae have the strongest staining for FGF9 although staining is seen in epithelial cells throughout the epithelium.

**Figure 5:**
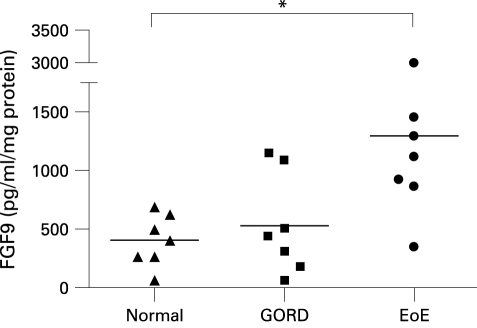
Fibroblast growth factor 9 (FGF9) protein in homogenates from oesophageal mucosal biopsies of patients with a normal oesophagus (triangles), gastro-oesophageal reflux disease (GORD, squares) or eosinophilic oesophagitis (EoE, circles), as detected by enzyme-linked immunosorbent assay (ELISA). Expressed as pg/ml of FGF9 per mg total protein as determined by Bradford assay. For each patient group, the horizontal line indicates the group average, *p<0.05.

**Figure 6:**
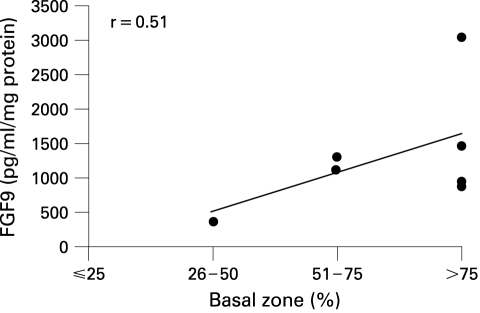
Fibroblast growth factor 9 (FGF9) concentration correlates positively with extent of basal zone hyperplasia (r = 0.51) in patients with eosinophilic oesophagitis (EoE). Basal zone hyperplasia (>25%) compared to FGF9 concentration (pg/ml/mg protein) as determined by enzyme-linked immunosorbent assay (ELISA), performed on separate oesophageal mucosal biopsies taken from an adjacent level.

Histopathological assessment of biopsies is summarised in table 2, with data presented as mean with the standard error. No evidence of inflammatory characteristics (increased intraepithelial inflammatory cells, basal zone >25% or elongated vascular papillae) were found in any samples from normal patients (n = 7). Patients with GORD (n = 7) had basal zone hyperplasia (>25%) or elongated vascular papillae and small numbers of eosinophils or other inflammatory cells in the epithelium. Most patients with EoE (n = 7) had marked eosinophilic inflammation and basal zone changes (table 2). Biopsies from patients with EoE (n = 7) had significantly increased basal zone changes, elongated vascular papillae and intraepithelial eosinophils, compared to normal patients (p<0.001). ELISA of supernatants from homogenised oesophageal mucosal biopsies, expressed per mg total protein, showed constitutive FGF9 protein with similar levels from normal and GORD patient biopsies and increased FGF9 from EoE patient biopsies (fig 5). FGF9 concentration in biopsies from patients with EoE correlated positively with basal zone hyperplasia (fig 6, r = 0.51).

**Table 2:** Assessment of inflammatory characteristics and fibroblast growth factor 9 (FGF9) concentration by diagnosis

Group	Maximum eosinophils/high-power field		Basal zone hyperplasia score*	Vascular papillae score*	FGF9 concentration (pg/ml/mg protein)*
	Number*	Range			
Normal	0 (0)	0	1 (0)	1 (0)	414.1 (89.0)
Gastro-oesophageal reflux disease	1.6 (1.4)	0–9	1.3 (0.2)	1.4 (0.2)	540.8 (173.0)
Eosinophilic oesophagitis	147.0 (36.7)	18–271	3.4 (0.3)	2 (0)	1296.0 (344.0)

*Results are given as the mean (SE).

## DISCUSSION

Eosinophilic oesophagitis is an emerging disorder, the cause of which is unclear. Basal hyperplasia is a common feature of oesophagitis and is particularly marked in biopsies from patients with EoE. Our results indicate that a synthetic MBP-peptide and polyarginine activate the CaSR on HET-1A cells, leading to increased FGF9 production. FGF9 causes HET-1A cellular proliferation and increased FGF9 is seen in biopsies of patients with EoE. Evidence of receptors for FGF9 suggests an autocrine action on oesophageal epithelial cells leading to proliferation. We have previously reported the presence of functional CaSR on HET-1A and that downregulation of the CaSR with siRNA (siRNA^CaSR^) transfection inhibited Ca^2+^-stimulated pERK, IL-8 secretion and intracellular Ca^2+^ mobilisation in HET-1A cells.^12^ Ca^2+^-stimulated BMP-2 production from colonic myofibroblasts^21^ and Ca^2+^-stimulated Wnt5a secretion from colon cancer cells^25^ have all been inhibited by transfection with siRNA^CaSR^. Therefore, the current experiments show that siRNA^CaSR^ abrogated polyarginine and MBP-peptide stimulation of FGF9 secretion, consistent with the interpretation that MBP acts through activation of the CaSR to stimulate FGF9 release. Treatment of these cells with a biologically active MBP-peptide^13^ increased FGF9 secretion in a manner comparable with addition of polyarginine.^26^ Because both the MBP-peptide and polyarginine increased FGF9 transcript and secretion, which was sensitive to RNA interference directed against the CaSR, our current results suggest that MBP may work as an agonist of the CaSR. Other eosinophil granule proteins such as eosinophil peroxidase, eosinophil cationic protein and eosinophil-derived neurotoxin also contribute to the pathophysiology of this disorder, but MBP was studied since it is the most abundant eosinophil product,^2^ ^3^ is well studied and mimics polyarginine, a known CaSR agonist. It would be interesting to utilise this model to investigate the effects of these other mediators on the CaSR and on FGF9 mediated proliferation.

Increased extracellular calcium concentrations did not increase FGF9 amplicons or stimulate FGF9 secretion from this oesophageal epithelial cell line, while polyarginine and MBP-peptide application did stimulate FGF9 release. We have used agonists of the CaSR in this cell line and have observed a variety of rapid and delayed biological events, which were abrogated by transfection of siRNA^CaSR^.^12^ Therefore, the current results suggest that MBP-peptide and polyarginine both activate the CaSR in HET-1A cells in a manner different to that of extracellular calcium. Other studies have shown that aromatic amino acids, when working as agonists of the CaSR, stimulate different signalling cascades to that of extracellular calcium.^27^ Additional studies are required to understand the mechanism of differential signalling of MBP and other agonists through the CaSR.

Regulation of proliferation in oesophageal epithelium is unknown. Genes related to the transforming growth factor β (TGFβ) superfamily, including bone morphogenetic proteins, are known to have altered mRNA transcript levels in proliferating cells.^28^ The genes for BMP-2, BMP-4 and noggin were examined since their level of expression is related to FGF9 activation,^19^ and proliferation in the gastrointestinal tract involving the CaSR.^21^ Transcripts of BMP-4 and noggin, were increased following FGF9 treatment. We postulate that FGF9 induces downstream signals promoting epithelial proliferation in the oesophagus, as it is known to do in other tissues.^19^ FGF9 also acts to downregulate the pro-apoptotic gene BMP-2, an antagonist of noggin, which may also contribute to increased epithelial cell number and hyperplasia during oesophageal inflammation. In summary, FGF9 increases transcription of genes that may affect cell number and the survivability of HET-1A cells.

In other tissues FGF9 acts following injury to promote mitogenic activity^16^ and wound healing^15^ in an autocrine and paracrine fashion.^16^ ^29^ Evidence that FGF9 secretion could result in autocrine activation leading to increased cell division in our experimental model was shown by transcript of receptors for FGF9 (fig 1C), altered transcript of downstream genes (fig 2A) and by proliferation of HET-1A cells as measured by BrdU and MTT assays (fig 2B,C). Increased BrdU incorporation in DNA is detected first, at 24 h, as cells progress through the S phase of the cell cycle;^30^ and MTT conversion is detected next at 48 h as a measure of metabolic activity related to the G_2_ and mitosis phases of the cell cycle.^31^ Proliferation was not observed at either 24 or 48 h after addition of MBP-peptide to HET-1A cells in culture (fig 3) indicating that the increased presence of FGF9 is due to an actual transcriptional phenomenon rather than an increase in overall cell number. Our experiments demonstrate that, in this model, only FGF9 induced proliferation of HET-1A cells.

FGF9 was increased in human oesophageal mucosal biopsies from patients with GORD and EoE. The most intense immunostaining was seen in basal epithelial cells (fig 4) in patients with EoE. FGF9 protein as detected by ELISA was increased in oesophageal mucosal biopsies from patients with GORD and EoE, with EoE reaching statistical significance compared with FGF9 concentrations in normal subjects (fig 5). These results, and evidence that MBP-peptide induces FGF9 in HET-1A cells, imply that increased FGF9 production is due to secreted MBP in active oesophagitis. The proliferative effects of FGF9 may then contribute to basal zone hyperplasia, a characteristic of oesophagitis. Basal zone hyperplasia correlated with FGF9 concentration (fig 6), but did not correlate with the number of eosinophils per HPF. Observation under light microscopy will detect intact eosinophils, whereas degranulated eosinophils, which have released MBP, will be more difficult to quantify. The variability in the FGF9 concentrations between individual patients, particularly those with EoE, is likely due to the varying amounts of soluble MBP present at different stages in the progression of EoE. The natural history of EoE is not well characterised and further studies are needed to understand the time course of EoE. We speculate that increased FGF9 levels would correlate closely with a stage of EoE following eosinophil degranulation.

**Figure 7:**
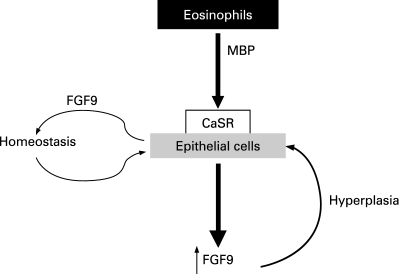
Schematic of proposed mechanism of calcium sensing receptor (CaSR)-mediated, major basic protein (MBP)-induced secretion of fibroblast growth factor 9 (FGF9) during oesophagitis. Constitutive FGF9 secretion likely plays a homeostatic role in oesophageal epithelium maintaining proliferation. During oesophagitis, activated eosinophils infiltrating the epithelium release MBP, selectively activating the CaSR, leading to increased FGF9 secretion. FGF9 subsequently has proliferative effects on the epithelium by autocrine action.

The above results suggest that, in EoE, activated eosinophils release MBP, causing activation of the CaSR on the surface of epithelial cells, which increases FGF9 production leading to basal cell hyperplasia, a feature of oesophagitis (fig 7). Therefore, autocrine and paracrine proliferative action of FGF9 in oesophageal inflammation may contribute to basal zone hyperplasia, a characteristic of oesophageal injury.
